# ﻿*Thiotrichasumpichi* sp. nov. – a new species of Thiotrichinae (Lepidoptera, Gelechiidae) from south-eastern Europe

**DOI:** 10.3897/zookeys.1173.105037

**Published:** 2023-08-01

**Authors:** Oleksiy V. Bidzilya, Peter Huemer, Ole Karsholt

**Affiliations:** 1 Institute for Evolutionary Ecology of the National Academy of Sciences of Ukraine, 37 Academician Lebedev str., 03143, Kyiv, Ukraine Institute for Evolutionary Ecology of the National Academy of Sciences of Ukraine Kyiv Ukraine; 2 Tiroler Landesmuseen Betriebsges.m.b.H., Natural History Collections, Krajnc-Str. 1, A-6060 Hall in Tirol, Innsbruck, Austria Tiroler Landesmuseen Betriebsges.m.b.H., Natural History Collections Innsbruck Austria; 3 Zoological Museum, Natural History Museum of Denmark, Universitetsparken 15, DK-2100 Copenhagen, Denmark Natural History Museum of Denmark Copenhagen Denmark

**Keywords:** Croatia, DNA barcoding, Greece, systematics, taxonomy, twirler moth

## Abstract

*Thiotrichasumpichi***sp. nov.** is described from Greece and Croatia. The systematic position of the new species within *Thiotricha* is discussed based on external and genitalia characters and from DNA barcodes of the mitochondrial COI gene (cytochrome c oxidase 1). Adults, details of external morphology, and male and female genitalia of the new species are illustrated.

## ﻿Introduction

Despite continuing efforts over many years, the taxonomy of European Gelechiidae is still incompletely covered and several genera and cryptic species groups await revision, probably with dozens of undescribed species (Huemer and Karsholt 2020; Huemer et al. 2020). One of the unresolved problems relates to an externally rather characteristic, small grey species with black markings from Greece and Croatia which has been known to the authors for many years. However, the generic and even tribal/subfamily assignment has remained uncertain. This taxon was first published as a genus and species close to *Caulastrocecis* Chrétien, 1931 by Huemer and Karsholt (2017), however, without detailed analysis of morphology and genetics. Extensive DNA barcoding of this species indicated a systematic placement near *Thiotricha* Meyrick, 1886. As a result of our detailed morphological study of this undescribed species we came to the conclusion that despite rather unusual admixture of external and genitalia characters, it shares several synapomorphies with the genus *Thiotricha*. In this contribution we therefore establish a new species of *Thiotricha* to accommodate this, locally rather abundant, species distributed in both the mainland and islands of Greece, and in Croatia.

## ﻿Material and method

The present paper is based on material from the following collections:

**KBE** Research collection of Kai Berggren, Kristiansand, Norway;

**NMPC**National Museum of the Czech Republic, Prague, the Czech Republic;

**SMNK**Staatliches Museum für Naturkunde, Karlsruhe, Germany;

**TLMF**Tiroler Landesmuseum Ferdinandeum, Innsbruck, Austria;

**ZMUC**Zoological Museum, Natural History Museum of Denmark, Copenhagen, Denmark;

**ZT** Research collection of Zdenko Tokár, Michalovce, Slovakia.

Male and female genitalia were dissected and prepared using standard methods (Huemer and Karsholt 2010). Male genitalia were spread using the unrolling technique as described by Pitkin (1986) and Huemer (1988). Pinned specimens and details of external morphology were photographed with a Canon EOS 5DS R DSLR camera (O. Bidzilya). Slide-mounted genitalia were photographed with a Canon EOS Rebel T5 DSLR camera mounted on an Olympus U-CTR30-2 trinocular head combined with a Carl Zeiss compound microscope (O. Bidzilya). For each photographed specimen, sets of 5–10 images were taken at different focal planes and focus-stacked using Helicon Focus 6 with the final image edited further in Adobe Photoshop CS5. Photographs of adults were taken with a Zeiss Stemi 508 KMAT stereo microscope, genitalia photographs with a Zeiss Axiolab 5 microscope, both adapted to an Olympus OM-D Mark III camera. Stacked photos were edited using Helicon Focus 4.8 and Adobe Photoshop 6.0 (P. Huemer).

The terminology of genitalia follows Lee et al. (2021) and Ponomarenko (2005).

Tissue samples (a single hind leg) of 26 specimens of *Thiotricha* were prepared for DNA barcoding (deWaard et al. 2008), a method which usually allows accurate species identification from a 658 base-pair long segment of the mitochondrial COI gene (cytochrome c oxidase 1), and successfully processed at the Canadian Centre for DNA Barcoding (CCDB, Biodiversity Institute of Ontario, University of Guelph). In addition, a single private sequence > 600 bp was made available to us in the Barcode of Life Data Systems (BOLD) (Ratnasingham and Hebert 2007; Ratnasingham 2018). Details including complete voucher data and images of our specimens can be accessed in the public dataset “New species of Thiotrichinae” (dx.doi.org/10.5883/DS-THIOSUMP) in BOLD. Sequences from the dataset were submitted to GenBank. All sequences were assigned to the Barcode Index Numbers (BIN), algorithm-based operational taxonomic units that provide an accurate proxy for the true species. BINs were automatically calculated for records in BOLD that comply with the DNA barcode standard (Ratnasingham and Hebert 2013). Follow-up species identification strictly followed available reference sequences in BOLD. Degrees of intra- and interspecific variation of DNA barcode fragments were calculated under the Kimura 2 parameter model of nucleotide substitution using analytical tools of BOLD systems v. 4.0. (http://www.boldsystems.org). Calculation of intraspecific distance was furthermore normalized with BOLD calculation tools to reduce bias in sampling at the species level. A Neighbour-Joining tree was constructed using the Kimura two-parameter model in MEGA7 (Kumar et al. 2016).

## ﻿Results

### ﻿Molecular analysis

**Figure 1.**
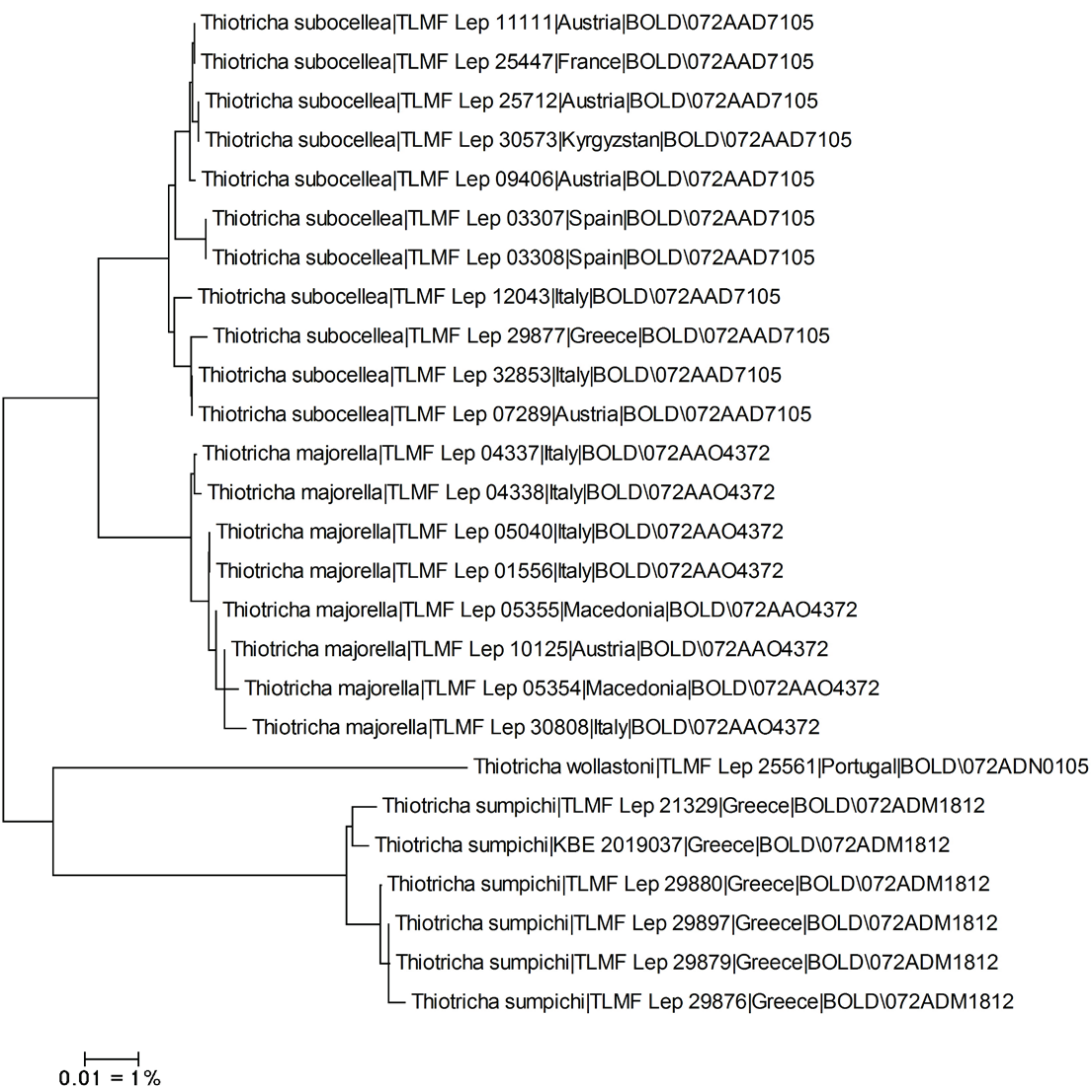
Unrooted Neighbour-Joining tree of European species of *Thiotricha* (Kimura 2 parameter, built with MEGA7 cf. Kumar et al. 2016), sequences (>600 bp) considered. Source: DNA barcode data from BOLD (Barcode of Life Database, cf. Ratnasingham 2018).

DNA barcoding resulted in a BIN concordant barcode fragment of >600 bp for 25 specimens and a single sequence of 413 bp for four European species of *Thiotricha*. Sequences revealed moderately low intraspecific, but significantly higher interspecific genetic distances (Table 1, Fig. 1). Whereas intraspecific divergence is unknown in *T.wollastoni* (Walsingham, 1884), mean divergence ranges from 0.52 to 0.8% in the other species. All species are attached to a unique BIN with the exception of *T.subocellea* (Stephens, 1834) where barcodes grouped in two BINs (Ratnasingham and Hebert 2013). In contrast, minimum interspecific divergence is 3.62% in *T.subocellea* and *T.majorella* (Rebel, 1910) whereas the distance of *T.wollastoni* and *T.sumpichi* sp. nov. to the nearest neighbour in *Thiotricha* is c. 10–11%. No DNA barcode is available for *T.coleella* since the only published sequence was based on a misidentified specimen (Huemer et al. 2020).

**Table 1.** Intraspecific mean K2P (Kimura 2 parameter) divergences (Mean Intra-Sp), maximum pairwise distances (Max Intra-Sp), nearest species, nearest neighbour (NN) (BOLD sequence ID) and distance to nearest neighbour (distances in %) in European species of *Thiotricha*.

Species	Mean Intra-Sp	Max Intra-Sp	Nearest species	NN	Distance to NN
*Thiotrichamajorella*	0.52	1.3	*Thiotrichasubocellea*	LEATE631-13	3.62
*Thiotrichasumpichi*	0.66	1.48	*Thiotrichasubocellea*	LEATE631-13	10.33
*Thiotrichasubocellea*	0.8	1.55	*Thiotrichamajorella*	PHLAB756-10	3.62
*Thiotrichawollastoni*	N/A	0	*Thiotrichasumpichi*	LECRT156-16	11.57

### ﻿Taxonomy

**Subfamily Thiotrichinae**

#### ﻿ Thiotricha sumpichi sp. nov.

Taxon classificationAnimaliaLepidopteraGelechiidae

466AD1B0-4847-5177-B620-FF519898328A

https://zoobank.org/25874C03-F319-4A9C-AE05-B4486CB0A215

Figs 2–8 , 9–13 , 14–20

##### Material examined.

***Holotype***: Greece • ♂; Peloponnes, Exochori, Viros Gorge; 36°54'23"N, 22°16'29"E; 470 m a.s.l.; 12–13 Sep. 2020; P. Huemer leg.; TLMF 2020-007; DNA barcode TLMF Lep 29897; TLMF.

***Paratypes***: Greece • 3 ♂♂, 3 ♀♀; same data as for holotype; DNA barcode TLMF Lep 29876; DNA barcode TLMF Lep 29879; DNA barcode TLMF Lep 29880; TLMF • 22 ♂♂, 29 ♀♀; Peloponnes, 3 km SW Poulithra; 37°05'36"N, 22°52'48"E; 450 m a.s.l.; 25 Sep. 2020; P. Huemer leg.; TLMF • 1 ♀; Peloponnes, above Pikiulionako/Mystras; 37°04'58"N, 22°20'31"E; 890 m a.s.l.; 22 Sep. 2020; P. Huemer leg.; TLMF • 1 ♂, 1 ♀; Peloponnes, Taygetos mts. E, Gorge Stavros-Kalamata; 37°04'46"N, 22°18'30"E; 780 m a.s.l.; 21 Sep. 2020; P. Huemer leg.; TLMF • 1 ♂; Peloponnes, Mani pen., SE Pyrrhichos; 36°38'10"N, 22°27'11"E; 360 m a.s.l.; 17 Sep. 2020; P. Huemer leg.; TLMF • 1 ♀; Peloponnes, Chelmos mts., above Zachlorou; 38°06'11"N, 22°09'16"E; 845 m a.s.l.; 27 Sep. 2020; P. Huemer leg.; TLMF • 1 ♀; Lakonia, 5 km S Monemvasia; 30 m a.s.l.; 7 Oct. 1977; G. Christensen leg.; ZMUC • 1 ♂; same collection data as for preceding; 9 Oct. 1977; G. Christensen leg.; gen. slide 88/20, Bidzilya; ZMUC • 3 ♀♀; same collection data as for preceding; 15 Sep. 1979; G. Christensen leg.; ZMUC • 1 ♂; same collection data as for preceding; 20 Sep. 1979; G. Christensen leg.; ZMUC • 1 ♂; same collection data as for preceding; ZMUC • 2 ♀♀; 27 Sep. 1979; ZMUC • 1 ♂; same collection data as for preceding; 13 Oct. 1979; G. Christensen leg.; ZMUC • 1 ♂; same collection data as for preceding; 31 Aug. 1980; G. Christensen leg.; ZMUC • 1 ♂; same collection data as for preceding; 12 Sep. 1982; G. Christensen leg.; ZMUC • 3 ♂♂, 1 ♀; same collection data as for preceding; 20 Sep. 1982; gen. slide 4416, 4417, H. Hendriksen; ZMUC • 1 ♀; same collection data as for preceding; 22 Sep. 1982; G. Christensen leg.; ZMUC • 1 ♀; same collection data as for preceding; 11 Oct. 1982; G. Christensen leg.; ZMUC • 1 ♀; same collection data as for preceding; 1 Oct. 1983; G. Christensen leg.; ZMUC • 1 ♀; same collection data as for preceding; 6 Oct. 1983; G. Christensen leg.; ZMUC • 2 ♂♂; 1 ♀; same collection data as for preceding; 7 Oct. 1983; G. Christensen leg.; ZMUC • 1 ♂; same collection data as for preceding; 11 Oct. 1983; G. Christensen leg.; ZMUC • 2 ♂♂, 1 ♀; same collection data as for preceding; 6 Oct. 1986; G. Christensen leg.; ZMUC • 2 ♂♂, 1 ♀; Lakonia, 7 km SW Monemvasia; 14 Sep. 1979; G. Christensen leg.; ZMUC • 2 ♂♂, 2 ♀♀; 22 Sep. 1979; G. Christensen leg.; gen. slide 184/21, O. Bidzylia, gen. slide 5539, R. Sutter; ZMUC • 1 ♂; 25 Sep. 1979; G. Christensen leg.; gen. slide 3722, O. Karsholt; ZMUC • 1 ♂; Lakonia, Monemvasia; 10 Oct. 1983; L. Gozmány leg.; ZMUC • 1 ♂; Crete; 1966; H. Reisser leg.; gen. slide 25/23, O. Bidzilya; SMNK • 1 ♂; Crete, Koutsounari; 4 Nov. 2004; 100 m a.s.l.; W. Ruckdeschel leg.; DNA barcode TLMF Lep 21329; TLMF • 1 ♂; Crete; Xekollimenos, Kirtomados; 27 Sep. 2001; ca. 40 m a.s.l.; W. Ruckdeschel leg.; DNA barcode TLMF Lep 21330; TLMF • 1 ♂; Crete; 3 km N Kapsodasos; 19 Sep. 2022; 650 m a.s.l.; P. Huemer leg.; TLMF • 3 ♂♂; Crete; 2 km NW Kolymbari, Astratigos; 1 Oct. 2016; 210 m a.s.l.; K. Larsen leg.; ZMUC • 1 ♀; Crete; 2 km N Kolymbari; 1–3 Oct. 2016; 10 m a.sl.;, leg. K. Larsen; ZMUC • 1 ♂; Crete; Chania, Kolymbari; 35°32'10"N, 23°48'4"E; 25 Sep. 2018; 5 m a.s.l.; K. Berggren leg.; KBE • 1 ♀; Kolymbari “W”; 35°32'10"N, 23°48'4"E; 102 m a.s.l.; 10 Sep. 2019; K. Berggren leg.; KBE • 1 ♂; 1 ♀; Crete; 1.7 km S Topolia; 2–3 Oct. 2016; 380 m a.s.l.; K. Larsen leg.; ZMUC • 1 ♂; 1 ♀; Prov. Etolia-Akanania, by Amvrakikos Bay, Katafourka; 8 Sep. 2008; 2 m a.s.l.; P. Skou leg.; ZMUC.

Croatia • 1 ♂; Zaostrog; 6 Sep. 2002; DNA barcode TLMF Lep 25652; Z. Tokár leg.; ZMUC; • 6 ♂♂; Zaostrog; 6 Sep. 2002; Z. Tokár leg.; gen. pr. Z. Tokár, ♂, 7978; ZT • 2 ♂♂, 1 ♀; Drvenik, Gornja Vala; 7–11 Sep. 2008; I. Richter leg.; DNA barcode TLMF Lep 25211; NMPC.

##### Diagnosis.

*Thiotrichasumpichi* sp. nov. can be separated externally from other European species of the genus by the grey forewing with brown longitudinal streaks. *Thiotrichacoleella* (Constant, 1885) from southern France remotely resembles *Th.sumpichi* sp. nov. by the forewing pattern, but differs in the presence of black spots rather than streaks. The male genitalia are characterized by having the valva distinctly broadened in the basal half, strongly edged posteromedial emargination of the vinculum and a short broadly rounded saccus. Other species of *Thiotricha* have unmodified or weakly broadened basally valva, gently edged posterior margin of the vinculum and short, usually subtriangular saccus. Segment VIII in the male is distinct by having a very short band-shaped tergum and large subtriangular sternum with broadened basal lobes separated by broadly rounded medial emargination, and narrow posterior portion with short apical incision. In *T.subocellea* segment VIII is similar in general, but the sternum is deeper bifurcated. Comparatively short (shorter than segment VIII) apophyses anteriores, antrum with narrow band of small triangular sclerites and long, slender longitudinal ridge-shaped signum are characteristic for the female genitalia of the new species. *Thiotrichasubocellea* has somewhat similar female genitalia, but apophyses anteriores are longer that segment VIII, the antrum lacks sclerites and the signum is crescent-shaped.

##### Description.

**Figures 2–8.**
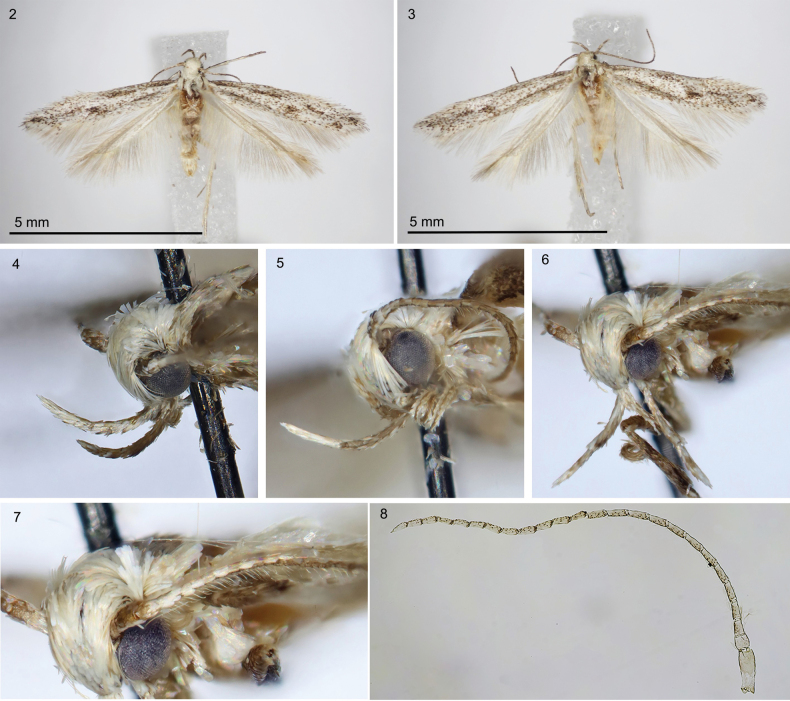
*Thiotrichasumpichi* sp. nov., Greece, paratypes **2, 3** adults, dorsal view **2** male **3** female **4–6** head, males **4, 5** lateral view **6** fronto-lateral view **7, 8** antenna, males **7** frontal view **8** slide 1/23, O. Bidzilya.

**Figures 9–13.**
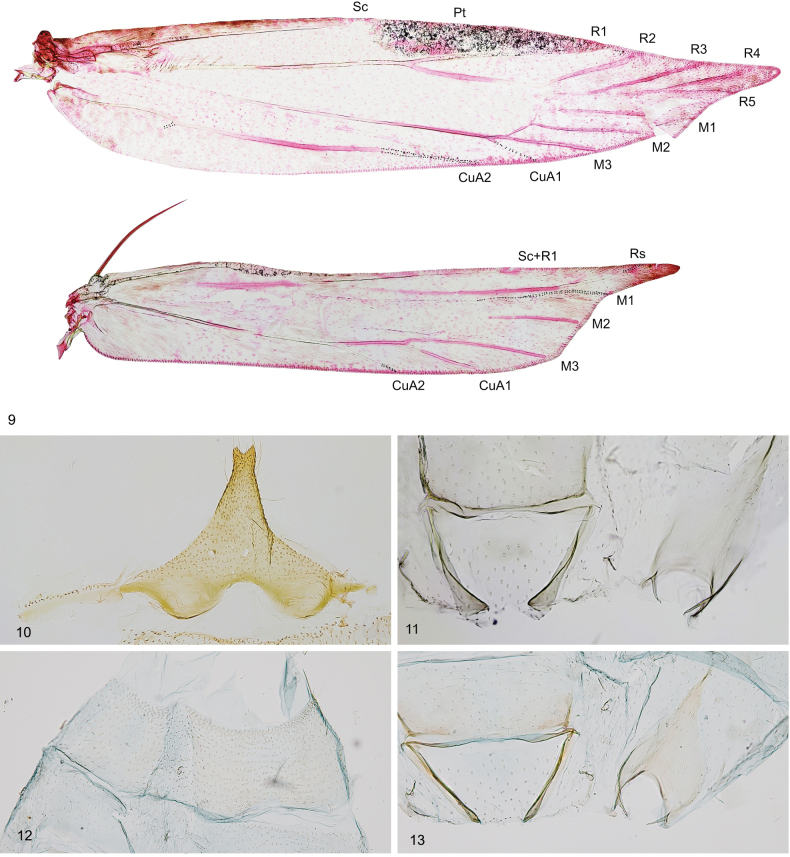
*Thiotrichasumpichi* sp. nov., Greece, paratypes **9** venation **10–13** abdominal segments **10** male segment VIII, tergum (left), sternum (right) **11** male segments I–II, tergum I–II (left), sternum II (right) **12** female segment VII, tergum (left), sternum (right) **13** female segments I–II, sternum II (left), tergum I–II (right).

**Figures 14–20.**
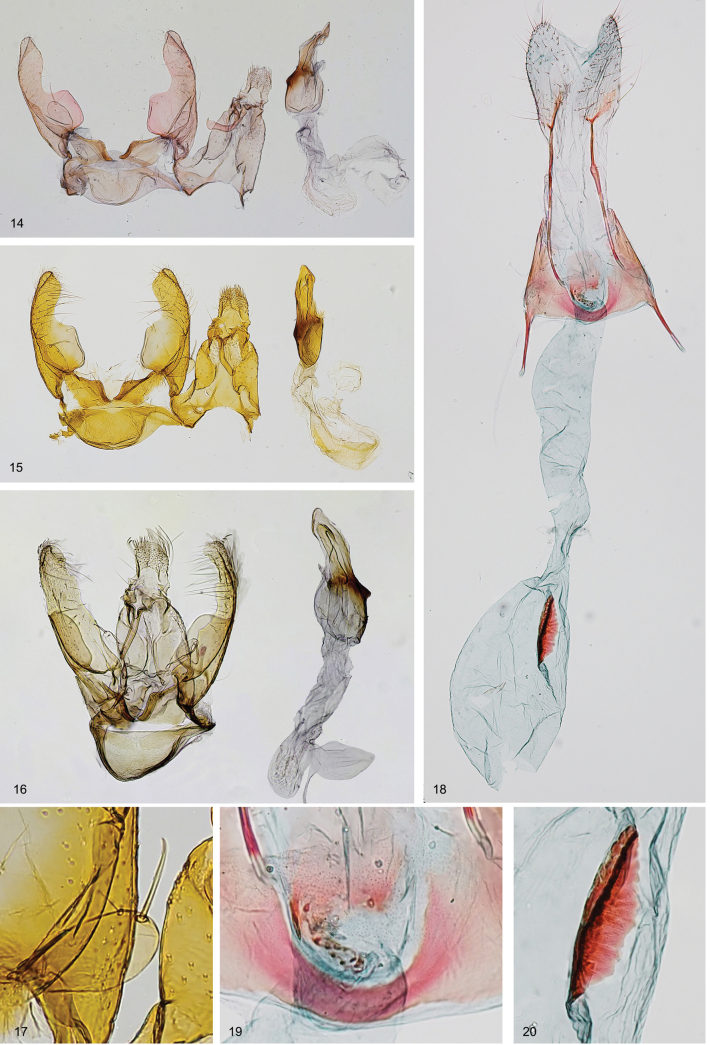
*Thiotrichasumpichi* sp. nov., Greece, paratypes **14–17** male genitalia **14** unrolled. Gen. slide 25/23, O. Bidzilya **15** unrolled. Gen. slide 88/20, O. Bidzilya **16** ventral view. Gen. slide 164/23, O. Bidzilya **17** valvella with apical bristle, enlarged. Gen. slide 88/20, O. Bidzilya **18–20** female genitalia **18** ventral view **19** antrum and ostium, enlarged **20** signum, enlarged.

***Head*** (Figs 4–6) white, smooth, ocelli absent; labial palpus white mottled with brown, strongly recurved, smoothly scaled, slender, 2.3 times as long as eye diameter, segment 2 as broad as and longer than segment 3; antenna without pecten, flagellomeres brown ringed with grey, shortly ciliate on underside in male (Figs 7, 8). ***Thorax*** and tegulae white. ***Forewing*** (Figs 2, 3, 9) elongated, with apex distinctly narrowed and pointed; venation (Fig. 9): Sc to about 1/2 of costa; R1 and R2 separated, to costal 2/3–3/4, pterostigma between Sc and R1; R3–R5 on common stalk, R3 and R4 to costa, R5 to termen; M1 and M2 parallel; CuA1 almost united at base to CuA2; A1+2 to 1/2 of dorsum; wingspan 9.5–10.5 mm, ground colour grey with brown suffusion along costal margin and in apical ¾, diffuse brown streak in fold, under costal margin to mid length, and from 2/3 of dorsal margin to wing apex, fringes white. ***Hindwing*** and fringes white, with costal margin arched and thickened to 1/2, then straight, parallel to dorsal margin, excavation distinct, apex narrow, pointed; venation (Fig. 9) with Sc+R1 to 1/2 of costa; Rs almost to apex; M1 weakly indicated, almost stalked with Rs, to termen; M2 to 1/2 of termen; M3 and CuA1 almost united at base; CuA2 to 2/3 of dorsum. ***Abdomen*. Male.** Tergum VIII very short, band-shaped (Fig. 10), sternum VIII (Fig. 10) subtriangular, broad anteriorly, with deep rounded anteromedial emargination and distinctly exceeding basal corners, distal portion narrow, posterior margin with shallow medial emargination, coremata absent; sternum II (Fig. 11) without group of sensory setae, apodemes short, free, venulae absent. In male genitalia (Figs 14–17) uncus broadly spatulate, twice as long as wide, densely covered with hairs in distal part; gnathos-hook about length of uncus, strongly curved at middle, apex weakly inflated with pointed tip; tegumen elongated with large lateral folds, parallel-sided, 2 times longer than wide, anteromedial excavation broadly rounded, extending to ¼ length of tegumen, lateral folds broad, curved inwards, transition to uncus distinct, large pedunculi sub-trianguar; valva long and stout, dorsal margin weakly bent, basal part with inwardly curved lobes two times broader than distal part, distal part as broad as uncus, with short subapical ridge, covered with short hairs, apex broadly triangular, exceeding apex of uncus, valvella ovate with apical bristle, extending to ¼ length of valva; vinculum short, band-shaped, posteromedial excavation deep, broadly cup-shaped, strongly edged; saccus short, broadly rounded; phallic tube as long as valva, with moderately inflated caecum, distal portion as long as caecum, with extension on ventral margin and with distinct trunk along dorsal margin, apex pointed or rounded, bulbus ejaculatorius about 1.5 times longer than phallic tube, with internal band-shaped sclerotization in anterior part.

**Female.** Tergum and sternum VII unmodified (Fig. 12), weakly emarginated posteriorly, tergum 1.5 times as long as broad, sternum 1.5 times as broad as long; sternum II (Fig. 13) without group of sensory setae, apodemes free, curved inwards, venulae absent. In female genitalia (Figs 18–20) papillae anales subovate, covered with short setae, apophyses posteriores about 2 times longer than segment VIII; apophyses anteriores shorter than segment VIII; tergum VIII evenly sclerotized, unmodified, trapezoidal, ostium opening indistinct, near anterior margin of sternum VIII, antrum cylindrical, U-shaped posteriorly, with narrow band of small triangular sclerites; ductus bursae moderately broad, with distinct transition to corpus bursae; corpus bursae ovate, membranous; signum long, 1/3 length of corpus bursae, with slender longitudinal ridge, in posterior portion of corpus bursae.

##### Etymology.

The new species is dedicated to Jan Šumpich (NMPC) in recognition of his contribution to the study of Palaearctic Lepidoptera.

##### Distribution.

Greece (south-western part, Crete), Croatia.

##### Molecular analysis.

BIN: BOLD:ADM1812 (*N* = 8). Intraspecific average distance within BIN is 0.72%, maximum distance is 1.14%. The minimum distance to the nearest congeneric neighbour, *T.subocellea*, is c. 10% (Table 1).

##### Biology.

**Figure 21.**
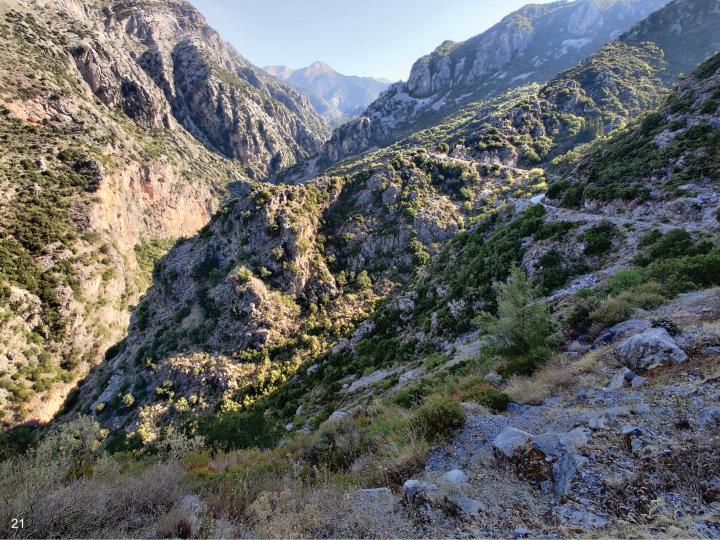
Habitat of *Thiotrichasumpichi* sp. nov.: Greece, Viros Gorge.

Host plant and immature stages unknown. Adults have been observed flying from late August to early November and were collected in large number at light (P. Huemer). The habitat is insufficiently known, but the species has been observed in a wide spectrum of different vegetation, mainly in open oak and maple forests intermixed with Mediterranean macchia (Fig. 21).

## ﻿Discussion

The species dealt with above has a restricted distribution in south-eastern Europe where it is locally common. The new species is small and grey, but the shape of the black markings on the forewings is distinctive. It has been known to lepidopteran specialists for several decades, but it could not be described until now because of uncertainty of its placement in the system of Gelechiidae. Instead, it was known by specialists as “*Caulastrocecis* sp.” (e.g., Huemer and Karsholt 2017), mainly based on external characters. During a recent revision of that genus (now *Ptycerata* Ely, 1910) (Bidzilya and Karsholt 2021) it became clear that the present species is not even distantly related.

We have been able to show that the new species fits into the subfamily Thiotrichinae. This is supported both by morphology and DNA barcode analyses, which, despite considerable divergences, group the new species nearest to European species of *Thiotricha* (Huemer 1993) (Fig. 1). Thiotrichinae are characterized by morphological characters such as a well-developed pterostigma in the forewing, a large sternum VIII and a reduced tergum VIII, valvella with a pair of digitate processes with one or a few apical setae, signum a plate (e.g., sickle-shaped, jar-shaped or occasionally missing), and the male antennae with rather long cilia (shorter in *Polyhymno* Chambers, 1874) (Karsholt et al. 2013; Gregersen and Karsholt 2022). The present species shares most of these characters.

Thiotrichinae is a moderately small subfamily of Gelechiidae, with about 180 described species in seven genera (Lee et al. 2021) and is most diverse in Asia. Since the description of the subfamily in 2013, they have been the subject of several studies (e.g., Lee et al. 2018, 2021; Kyaw et al. 2019; Lee and Hayden 2019). Lee et al. (2021) presented a phylogeny of the subfamily with a revision of the generic classification based on molecular and morphological analyses. Based on that we are able to suggest a placement for the new species in *Thiotricha*.

Thiotrichinae currently comprises seven genera: *Thiotricha*, *Polyhymno* Chambers, 1874, *Macrenches* Meyrick, 1904, *Calliprora* Meyrick, 1914, *Pulchrala* Lee & Li, 2021, *Tenupalpa* Lee & Li, 2021 and *Palumbina* Rondani, 1876 (Lee et al. 2021). *Thiotrichasumpichi* sp. nov. differs from these genera by the wide basal lobe of the valva in the male genitalia and by the lack of pecten on the antennal scape which is characteristic for other Thiotrichinae genera. Despite these autapomorphies, the new species shares with *Thiotricha* the following characters: (1) antennal ciliation in the male; (2) absence of Rs4 vein in the forewing; (3) male sternum VIII broadened at the base with a tendency to be bifurcate apically; (4) vestigial coremata; (5) elongate slender signum; and (6) modified base of the valva. We therefore tentatively include the new species in *Thiotricha*. However, *Thiotricha* is rather diverse morphologically and in need of revision. Several DNA barcode lineages require additional molecular data and extensive analysis of morphology (Fig. 1). In a phylogeny based on both morphology and molecular data of several taxa presented by Lee et al. (2021)*Thiotricha* clusters into two main groups. One includes the type species of the genus *T.thorybodes* Meyrick, 1886 from New Zealand. In the other group we find the European *T.subocellea* (Stephens, 1834) which is type species of the genus *Reuttia* Hofmann, 1898 and all other continental European congeners (Huemer 1993). It is beyond the scope of the present study to evaluate if *Thiotricha* should be split into several genera.

## Supplementary Material

XML Treatment for Thiotricha sumpichi

## ﻿Additional information

### Author ORCIDs

Oleksiy V. Bidzilya https://orcid.org/0000-0001-9243-2481

Peter Huemer https://orcid.org/0000-0002-0630-545X

Ole Karsholt https://orcid.org/0000-0002-6969-2549

### Data availability

All of the data that support the findings of this study are available in the main text or Supplementary Information.
